# Does Universal Screening for Gestational Diabetes Mellitus Improve Neonatal Outcomes in a Socially Vulnerable Population: A Prospective Study in French Guiana

**DOI:** 10.3389/fendo.2021.644770

**Published:** 2021-05-21

**Authors:** Loic Leonco, Hatem Kallel, Mathieu Nacher, Liliane Thelusme, Maryvonne Dueymes, Raoudha Mhiri, Marie Laure Lalanne-Mistrih, Nadia Sabbah

**Affiliations:** ^1^ Department of Endocrinology and Metabolic Diseases, Centre Hospitalier Andrée Rosemon, Cayenne, French Guiana; ^2^ Department of Intensive Care, Centre Hospitalier Andrée Rosemon, Cayenne, French Guiana; ^3^ Clinical Investigation Center, West Indies, French Guiana (INSERM CIC 14 24), Centre Hospitalier Andrée Rosemon, Cayenne, French Guiana; ^4^ Department of Biology, Immunology and Parasitology, Centre Hospitalier Andrée Rosemon, Cayenne, French Guiana; ^5^ Department of Gynecology and Obstetrics, Centre Hospitalier Andrée Rosemon, Cayenne, French Guiana; ^6^ Department of Nutrition (UTDN-CSO), Centre Hospitalier Universitaire de Guadeloupe, Pointe à Pitre, Guadeloupe, France; ^7^ EA3593, Amazon Ecosystems and Tropical Diseases, Université de Guyane, Cayenne, French Guiana, France

**Keywords:** French Guiana, gestational diabetes mellitus, macrosomia, poverty, systematic screening procedure

## Abstract

**Aims/Introduction:**

French Guiana has a high prevalence of metabolic diseases, which are risk factors for gestational diabetes mellitus. Despite routine screening for gestational diabetes, treatment is still challenging because of health inequalities and different cultural representations of disease and pregnancy. This study was conducted to assess the role of early and universal GDM screening on obstetrical and neonatal complications in a socially deprived population.

**Materials and Methods:**

A prospective study was conducted, in the level III maternity in French Guiana. Of 2136 deliveries, 223 had gestational diabetes mellitus, 110 of whom were followed-up for 6 month to detail their social and laboratory parameters.

**Results:**

The prevalence of gestational diabetes in French Guiana (Cayenne Hospital) was estimated at 10.3%. The study population was very precarious with 70% of patients on welfare (universal health coverage or state medical assistance). The following obstetrical complications were observed: cesarean delivery (32%), history of miscarriage (26%) and preeclampsia (7.4%). Nevertheless, neonatal complications were rarely present and included hypoglycemia (2.8%) and macrosomia (2.8%).

**Conclusion:**

In French Guiana, gestational diabetes mellitus is very common. However, in a context of widespread poverty and diverse cultural representations, universal screening and monitoring limited the risk of macrosomia.

## Introduction

French Guiana—a French territory between Brazil and Suriname—has one of the highest birth rates in Latin America and in France [https://www.insee.fr/fr/statistiques/3309060]. There is a very high prevalence of preterm delivery and preeclampsia (1–3). In addition, the prevalence of obesity and diabetes in French Guiana are very high (respectively 17% (4) and 10% (5), notably among women). In a context of massive immigration, geographical isolation, and low medical density, social inequalities of health are widespread (6), notably for obstetrical problems (2). The Level III maternity (managing high risk pregnancies) in French Guiana is located in Cayenne Hospital. Patients from isolated areas and, especially, patients with gestational diabetes mellitus (GDM) are usually transferred to the Level III maternity unit from the 8th month of pregnancy onward for monitoring, due to the geographic distances. The risk factors most often associated with GDM include being overweight (body mass index [BMI] >25), age (>35 years), history of GDM, familial diabetes, and ethnicity (7, 8). Obstetrical and neonatal complications of GDM include macrosomia, preeclampsia, prematurity, shoulder dystocia, neonatal hypoglycemia, and cesarean delivery (8). The diagnostic and screening criteria for patients with GDM vary between different countries. In 2011, Vignoles et al. reported a GDM prevalence of 5.1% in Guadeloupe (French overseas department) using the World Health Organization (WHO) criteria (7, 9); while in Mainland France the GDM prevalence was found between 5 and 8%, but a single center study detected during a systematic screening a prevalence of 14% based on the International Association of Diabetes and Pregnancy Study Groups (IADPSG) criteria (10). Some studies have found differences in GDM prevalence that are linked to ethnicity; In 2012, a study conducted in the United States by Kim et al. found variable GDM prevalence, with Pacific Islanders being at a relatively high risk (9.9%) (11).

Unlike in mainland France, since 2015 screening for GDM is routinely undertaken. This was implemented by the perinatal network –a network of obstetricians and midwives disseminating best practices in French Guiana— in order to have a standardized attitude because evaluating risk factors for gestational diabetes is challenging in the most isolated areas of the territory, and because the prevalence of obesity and diabetes are among the greatest in France, notably in women younger than 35 years (5). The selection criteria for screening for GDM in France are not adapted to French Guiana, which has many different ethnic groups, as described in the study by Cosson et al. (12)

Although the incidence of macrosomia and caesarian section in GDM are higher in low and middle-income countries, the literature is scarce about the specific role played by poverty and social deprivation in obstetrical and neonatal complications in gestational diabetes mellitus. Furthermore, the impact of universal screening, early treatment and monitoring of GDM is unknown in particular in the most deprived populations.

The principal objective of this cross-sectional study is to analyze the role of early and universal GDM screening on obstetrical and neonatal complications in a socially deprived population.

Furthermore, it is necessary to highlight the usefulness of systematic screening and hospitalization in the obstetrical wards for women with obstetrical complications or difficult access to care from the 8^th^ gestational month. The geographic isolation and poverty of the population in this region makes it essential to implement a locally appropriate, graduated care policy for the treatment of GDM.

## Materials and Methods

### Study Site

This single-center, prospective descriptive study took place at Cayenne Hospital over a 6-month period (December 1, 2017 through June 1, 2018) with continuous data collection. Patients were admitted to the adult diabetes outpatient, the short-stay unit, or Cayenne Hospital’s maternity ward for complicated pregnancies. There are 3 public hospitals in French Guiana, one in each main coastal city and two private hospitals. The healthcare infrastructure also comprises 17 health centers and 15 maternal and child-care centers. There is only 1 Level III maternity in French Guiana located in Cayenne hospital. Because of geographic distances, patients from isolated areas –especially patients with diabetes— are usually transferred to the Level III maternity unit from the 8th month of pregnancy onward.

### Inclusion and Exclusion Criteria

The inclusion criterion was a diagnosis of Gestational Diabetes Mellitus according to the International Association of Diabetes and Pregnancy Study Groups (IADPSG) criteria (13) in female patients (age 18–46 years) hospitalized at Cayenne Hospital during the study period. The exclusion criteria were: refusal to provide written informed consent, preexisting type 1 or 2 diabetes, or failing to meet the inclusion criteria.

### Definition of GDM

GDM was confirmed when fasting glycemia reached or exceeded 92 mg/dL (5.1 mmol/L). Furthermore, pregnant patients undergo an oral glucose tolerance test with a 75-g glucose load between 24 and 28 gestational weeks, and are diagnosed as GDM if at least one plasma glucose measurement reaches or exceeds the following thresholds: glycemia at t0 of >92 mg/dL (5.1 mmol/L) and/or 1-hour plasma glucose >180 mg/dL (10.0 mmol/L) and/or 2-hour plasma glucose >153 mg/dL (8.5 mmol/L) (14, 15).

### Study Conduct and Judgment Criteria

**Figure 1:**
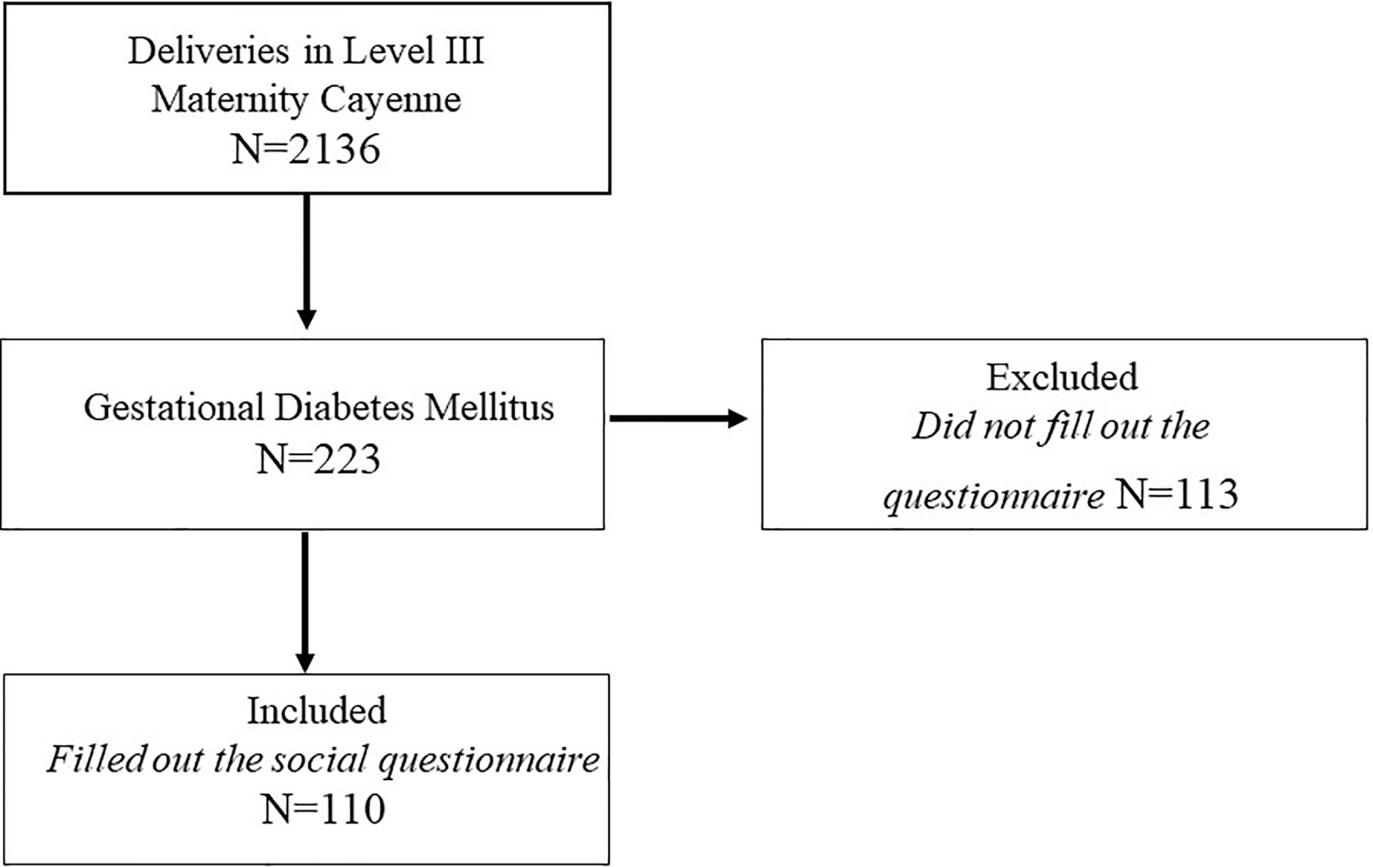
Flow-chart Gestational Diabetes Mellitus patients included during the study period.

The total number of deliveries during the study period was 2136. Among these, 223 patients were hospitalized and diagnosed with GDM during the study period (data collected by the Department of Medical Information [DIM]). Prevalence of GDM in Cayenne was hence obtained by dividing the number of GDM cases by the number of deliveries during the study period. Among the 223 women with GDM, a subsample of 110 women seen at the diabetology day hospital –where they had the time, assistance and if needed translation services to fill-in the social questionnaire—were enrolled for further data collection and laboratory testing (**Figure 1** and **Table 3**). Laboratory blood test were taken on the day of enrollment. When diagnosed with GDM, patients are referred to our outpatient diabetology unit, which allows them to access health education, dietitian and diabetologist consultations; they are then monitored externally by a nurse, in particular when they receive insulin, and by a midwife trained in diabetes management. They are followed (consultation) every 15 days by education nurses and are required to send their daily blood sugar self-control to the diabetology department.

Clinical and laboratory data as well as obstetrical and neonatal complications were collected after the first medical contact from electronic hospital and laboratory records. To estimate precariousness, a binary variable was created to differentiate between patients with a monthly income >1000 or <1000 euros, a threshold chosen to fit the definition of the poverty line in French Guiana. A second variable was created to differentiate between patients with monthly income >500 and <500 euros, a threshold chosen to look for extreme poverty (16). Finally, unemployed patients were differentiated from patients who were students or employed.

In addition, we differentiated patients who received state-sponsored medical assistance (Aide Médicale Etat [AME]), which is an emergency assistance for undocumented foreign patients without health insurance. Moreover, we categorized patients receiving universal health coverage (Couverture Maladie Universelle [CMU]; low-income patients (French or legal residents) receiving social insurance benefits) and other patients with normal health insurance.

### Statistical Analysis

Quantitative variables were expressed as median and interquartile range. Qualitative variables were expressed as frequencies and percentages. The means were compared using Student’s *t*-test. Qualitative variables were compared with the chi-square test. Variables related to the outcome on univariate analysis were subsequently analyzed in a multivariate logistic regression model. A *p*-value <0.05 was considered significant. All statistical analyses were conducted using SPSS^®^ software, version 24.

## Results

Overall, during the 6-month study period, the medical information system showed that GDM prevalence was 10.3% (223/2136), (95% confidence interval (95%CI) = 9.17-11.8%). Among the subsample who received the social questionnaire, the mean age was 32.8 years [21-45 years], and the mother tongue was mostly a foreign language and 40% of the women were single; Most were unemployed and lived under poverty line (<1000 euros per month) and half had a monthly income <500 euros; 70% benefited from universal health insurance (CMU) or state medical aid (AME). The socioeconomic and language characteristics are described in **Table 1**.

**Table 1:** Medical and social criteria.

	Number *n*	Percentages (%)
**Risk factors for gestational diabetes (GDM)**		
Family history of diabetes	49	44.5
History of GDM	18	16.5
History of macrosomia	11	10
Age > 35 years	55	50
Body mass index > 25 kg/m² (before pregnancy)	26 *	74
**Socioeconomic data**		
Unemployed	76	69
CMU/CMUc/AME	75	68
3 meals per day	93	85
Income < 1000 euros per month	64	58
**Cardiovascular risk factors**		
History of high blood pressure	10	9
Dyslipidemia	1	1
**Obstetric history**		
Miscarriages	26	24
Abortion	28	25.5
Cesarean	13	12
Ectopic pregnancy	2	2
Abortion for medical reasons	2	2
**Spoken languages**		
French	83	75
Brazilian Portuguese	10	9
Haitian Creole	49	45
Guianese Creole	26	23.6
English	10	9
Spanish	12	11
Bushinengue	5	4.5

Macrosomia: newborn weighing more than 4kg, CMU, Couverture maladie universelle (universal health coverage).

CMUc Couverture maladie universelle complémentaire (complementary universal health coverage) AME, Aide médicale d’état (state medical assistance).

*on 35 available data.

The overall mean number of pregnancies was 4.1 per woman and the mean number of children was 2.3 (range 1-8). Patients with a history of multiple abortions were found to be at significantly higher risk for obstetrical complications than those without abortions (**Table 3**). Obstetrical history is described in **Table 1**. Half of the women reported a family history of diabetes, and 18 (16.5%) said they previously had GDM. Two-thirds of patients (67%) had a history of one or more obstetrical complications. Many women had a history of obstetrical complication—most often in their country of origin (80% in Haiti).

The mean gestational duration was 38 weeks and 1 day. Fifty percent of patients were on a diet alone and 50% were on insulin (detemir or glargine).

Present obstetric complications were: cesarean section (32.7%), prematurity (12.7%), fetal heart arrhythmia (11.2%), pre-eclampsia (7.4%), premature membrane rupture (5.6%), and only 3 (2.8%) macrosomia (more than 4kg) (**Table 2**). Seventeen newborns had complications (detailed in **Table 2**), the most frequent was respiratory distress. One neonatal death, and one loss to follow-up were reported. The mean infant length, head circumference (HC), and weight were 48 cm, 34.1 cm, and 3086 g, respectively.

**Table 2:** Obstetrical and neonatal complications.

	Number *n*	Percentages (%)
Obstetric complications	31	28.9
Cesarean	36	32.7
Macrosomia	3	2.8
Premature membrane rupture	6	5.6
Retroplacental hematoma	2	1.9
Uterine rupture	2	1.9
Preeclampsia	8	7.4
Fetal heart arrythmia	12	11.2
Prematurity	14	12.7
Neonatal complications	17	15.5
Hypoglycemia	3	2.8
Respiratory distress	13	12.3
Maternal-fetal infection	8	7.4
Patent ductus arteriosus	2	1.9
Shoulder dystocia	1	0.9

Nearly 77% of women had anemia (hemoglobin <11.5 g/dL; n=82), and 6 patients (5.7%) had thrombocytopenia.

The glycated hemoglobin (HbA1c) and fructosamine assays were abnormal (>5.9% and >285 µmol/L, respectively) in 20 (20.8%) and 38 (39%) patients, respectively, of the 96 patients who were tested. Eighty-four patients underwent microalbuminuria assay, 28.5% had abnormal results (>20 mg/L); of these 24 patients, 2 (8%) had a history of chronic hypertension and 2 (8%) preeclampsia.

Patients with a history of multiple abortions were found to be at significantly higher risk for obstetrical complications than those without abortions (**Table 3**). We found no significant differences with regard to the occurrence of obstetrical complications in patients with a history of GDM, familial diabetes, macrosomia, preeclampsia, or abnormal values for HbA1c, fructosamine or microalbuminuria (**Table 3**).

**Table 3:** Bivariate analysis of socioeconomic and medical data in relation to onset of obstetric or neonatal complications.

	Obstetric complications n (%)			Neonatal complications (%)		
	Yes	No	p-value	Yes	No	p-value
**Socioeconomic data**						
AME	3 (15)	17 (85)	0,127	3 (15)	17 (85)	0.979
CMU/CMUc/AME	21 (28.7)	52 (71)	0.945	13 (17.8)	60 (82.2)	0.206
CMU/CMUc/AME + Haitian creole	8 (17.7)	37 (82.2)	0.03*	8 (17.8)	37 (82.2)	0.464
French-speaking	29 (36.2)	51 (63.7)	0.004*	14 (17.2)	67 (82.7)	0.211
Haitian creole-speaking	9 (18)	41 (82)	0.019*	8 (16)	42 (84)	0.748
English-speaking	6 (60)	4 (40)	0.023*	0 (0)	10 (100)	0.166
Income < 1000 euros	18 (28.5)	45 (71.4)	0.939	10 (62.5)	6 (37.5)	0.915
Unemployed	19 (26)	54 (73.9)	0.325	11(68.7)	5 (31.2)	0.864
**History and risk factors**						
History of gestational diabetes (GD)	3 (16.6)	15 (83.3)	0.18	2 (11.1)	16 (88.9)	0.592
Family history of diabetes	16 (33.3)	32 (66.6)	0.256	7 (14.5)	41 (85.4)	1
Macrosomia	2 (18.1)	9 (81.8)	0.418	1 (9)	10 (90.9)	0.601
History of abortion	13 (46.4)	15 (53.5)	0.015*	2 (20)	8 (80)	0.979
History of preeclampsia	5 (50)	5 (50)	0.113	4 (14.2)	24 (85.7)	0.586
**Lab tests**						
Fructosamine (> 285 µmol/L)	4 (11.4)	31 (88.5)	0.024*	2 (5.6)	34 (94.4)	0.072
Microalbuminuria (> 20 mg>/L)	7 (29.1)	17 (70.8)	0.517	6 (25)	18 (75)	0.082
HbA1c (> 5.9%)	4 (20)	10 (80)	0.207	3 (15)	17 (85)	0.160

CMU, Couverture maladie universelle (universal health coverage). *significant (p-value < 0.05).

### Important Negative Results

The cross tabulation of socioeconomic data with obstetrical complications clearly showed a higher risk of complications in French-speaking and English-speaking patients (**Table 3**). Patients speaking Haitian Creole and/or with universal health coverage, complementary universal health coverage, or state medical assistance had significantly fewer obstetrical complications (**Table 3**). There were no significant differences for age, marital status, type of coverage, monthly income, or occupation (**Table 3**).

No significant differences were found with regard to neonatal complications based on the patient’s type of coverage, age, monthly income, occupation, or a history of GDM, familial diabetes, macrosomia, or preeclampsia. Abnormal HbA1c, microalbuminuria, or elevated fructosamine were not associated with a significantly higher rate of neonatal complications. Boys had significantly fewer neonatal complications (*p*=0.01) (**Table 3**).

## Discussion

We found that the prevalence in Cayenne (one of the two maternity hospitals in the area) of gestational diabetes was high at 10.3% of pregnancies. Despite variations between studies with different diagnostic criteria, the observed prevalence is twice the GDM prevalence in mainland France (hospital database) (10, 14, 17, 18). As in the West Indies and Reunion Islands, prevalence of metabolic diseases is double of what is observed in mainland France (4, 5, 19). Although, there are no published studies focused on GDM prevalence and characteristics in French Guiana, a general study on hospital deliveries between 2013 and 2014 observed 0.5% of preexisting diabetes, and a 4.7% of gestational diabetes (615 on 12983 births) but probably underestimated because less screened (realized before the implementation of systematic screening, in 2015) (2). There are only two maternity units where patients with gestational diabetes or type two diabetes give birth, which limits selection bias.

We showed that 81 patients (73.6%) had one or more risk factors for developing GDM. The main known risk factors were: age > 35 years (50%), family history of diabetes in a first or second-degree relative (50%), history of GDM (16%) and ethnic characteristic (black, and hispanic) (20). Several studies demonstrated the role of maternal age as a risk factor within the framework of GDM screening (7, 8, 21). The mean age in our study was indeed similar to reports from France and worldwide (13, 14). Although the risk of GDM in women older than 35 is often underestimated (21), universal screening in French Guiana –the only French territory to do so (22)—allows to avoid missing cases of GDM.

Sixty percent of women with GDM in Cayenne had a history of obstetrical complications, 10% of macrosomia, and we highlighted a frequent history of miscarriages in comparison with other French territories, much higher than the 12% reported in mainland France (23, 24). This high frequency miscarriages, especially in Western French Guiana (43%), emphasizes the challenges associated with monitoring difficult pregnancies.

Despite the frequent history of a variety of adverse obstetrical outcomes, the initial interview showed that most of these complications in fact occurred in their country of origin (most often Haiti). The literature on obstetrical and neonatal complications in Haiti is very poor, but there is a significant morbidity and mortality rate, particularly related to acute complications during pregnancy (25, 26).

Health issues in French Guiana are shaped by intense immigration and poverty, a shortage of health professionals, and logistical constraints to ensuring continuity of care, particularly in isolated areas. Most women included in the study came from a foreign country.

Of the 110 patients having answered the social questionnaire, nearly 1/3 had obstetrical complications. The rate of cesarean delivery was higher for pregnancies complicated by GDM (36%) than for normal pregnancies (27) or among women with GDM in mainland France (27.8%) (8). Contrarily to what has been reported elsewhere, there was no difference in cesarean section rate between precarious and non-precarious women (28).

All complications cannot be attributed to GDM, in particular pre-eclampsia, because the causes are often multifactorial, and several metabolic problems and hypertension are intertwined before pregnancy (2).

Half of patients in our study were receiving insulin. The high prevalence of prepartum obesity in French Guiana may partially explain the greater proportion of GDM patients treated with insulin relative to mainland France. This frequent insulin prescription is consistent with reports that the proportion of patients treated with insulin increased with the patient’s BMI (18). Moreover, insulin may be the only solution in difficult social contexts where dietary modifications –the first treatment of choice for GDM—is financially impossible (anti diabetic drugs as being completely covered by the health insurance).

GDM increases obstetrical and neonatal complications and early screening and management is necessary to prevent them (29–31). The biological markers for determining GDM fructosamine and, more rarely, HbA1c were elevated in 39% and 11% of patients, respectively. No significant difference was shown for HbA1c in relation to the risk of onset of obstetrical and/or neonatal complications. The interest of the HbA1c assay during pregnancy is controversial but most agree that its interest is limited compared to screening according to the IASPDG criteria (32); indeed, it represents the average blood sugar level from the previous 2 or 3 months, and it increases during iron deficiency with or without anaemia (33). The cut off corresponding to the level of HbA1c which must be considered as predictor of adverse obstetric outcomes during pregnancy has not been established, and varies between 5.55 (34) and 5.9% (35). Moreover, fructosamine is not a reliable biological marker in screening for GDM because it lacks sensitivity (36, 37). Very few studies have evaluated its connection with neonatal outcomes, and the results are inconsistent (17, 38). Nevertheless, recent studies showed that fructosamine is a better predictor of birthweight and large-for-gestational age infants than HbA1c (17).

Several studies showed that microalbuminuria at the end of the second trimester of a nondiabetic pregnancy could increase the risks of preterm labor, preeclampsia, intrauterine growth restriction, and premature membrane rupture (39–41). In our study however microalbuminuria was not associated with higher risk of neonatal or obstetrical complication.

Fifteen and a half-percent of newborns from mothers with GDM had one or more complications. The commonest complications were respiratory distress and feto-maternal infections; Hypoglycemia, macrosomia and shoulder dystocia were very rare. GDM treatment decreases the risk of macrosomia, large for gestational age births, and shoulder dystocia but not neonatal hypoglycemia, preterm births, pre-eclampsia, or caesarean section (27). Macrosomia has been reported in 15% of newborns from a cohort of GDM mothers in France, with a 3.6% rate of respiratory distress at birth (14). In contrast, in French Guiana, the prevalence of macrosomia (2.6%), corrected for age and sex, was low, despite frequent social precariousness (42). In Cuba, Cruz et al. found macrosomia rates of 10% to 20% (7). This difference between our study and the prevalence of macrosomia in neighboring countries or in South America, where precariousness is also frequent, is probably linked with the routine hospital admission of high-risk pregnancies in French Guiana during the last 2 months and/or weekly follow-up in multidisciplinary consultations for patients with easy mobility.

Many of our patients had previously given birth in a country with limited healthcare resources and difficulties in access to care, and screening was late. In French Guiana, these women benefitted from stringent monitoring because they are specifically at high risk of complications. In Caribbean and Latin American countries, complications are higher than in French Guiana despite comparable precariousness and gestational diabetes (43). In 2015, a French study carried out on a precarious population, revealed a more precocious onset of gestational death, more shoulder dystocia and more large for gestational age infants (44). However, women from Surinam or Guyana –but not Haiti—had more obstetrical complications than other patients; Perhaps this is due to language difficulties, cultural representations and social obstacles to regular care and monitoring.

Although the prevalence estimation had a narrow confidence interval, our descriptive study’s weakness was linked to its small sample size and lack of statistical power. Moreover, there was no postpartum follow-up, which is an essential part of metabolic screening. Patients were contacted again after delivery, but postpartum data collection was difficult because, most patients had not had their fasting glucose levels checked or could not be reached, which is another characteristic of vulnerable populations in French Guiana.

## Conclusion

Gestational diabetes is very common in French Guiana, where screening is universal. The population in our study was poor and culturally diverse. Cesarean delivery and preeclampsia were the main obstetrical complications identified, while the main neonatal complications were prematurity and respiratory distress. The rate of macrosomia, however, was very low in our population. GDM screening and intensive follow-up of psychosocial deprivation pregnancies in French Guiana is a major advantage, and clearly makes it possible to limit common GDM complications.

## Data Availability Statement

The raw data supporting the conclusions of this article will be made available by the authors, without undue reservation.

## Ethics Statement

The studies involving human participants were reviewed and approved by the General Data Protection Regulations (GDPR) and in compliance with both French and European regulations (EU 2016/679), and reported to the National Institute of Health Data (INDS) under registry number MR3014270220. The patients/participants provided their written informed consent to participate in this study.

## Author Contributions

LL, HK, MN, and NS participated in study protocol development, data collection, and data analysis and co-authored the manuscript. ML-M participated in data collection writing, and analysis. LT, MD, and RM participated in data collection. All authors contributed to the article and approved the submitted version.

## Conflict of Interest

The authors declare that the research was conducted in the absence of any commercial or financial relationships that could be construed as a potential conflict of interest.

## Abbreviations

AME, Aide médicale d’état (state medical assistance); CGSS, Caisse générale de sécurité sociale (general social security fund); CMU, Couverture maladie universelle (universal health coverage); CMUc, Couverture maladie universelle complémentaire (complementary universal health coverage); DIM, Department of Medical Information; GDM, Gestational Diabetes Mellitus; HbA1c, glycated hemoglobin; IADPSG, International Association of Diabetes and Pregnancy Study Groups; INSEE, Institut National de la Statistique et des Etudes Economiques (French National Institute of Statistics and Economic Studies); WHO, World Health Organization.
